# Blue light-dependent human magnetoreception in geomagnetic food orientation

**DOI:** 10.1371/journal.pone.0211826

**Published:** 2019-02-14

**Authors:** Kwon-Seok Chae, In-Taek Oh, Sang-Hyup Lee, Soo-Chan Kim

**Affiliations:** 1 Department of Biology Education Kyungpook National University, Daegu, Republic of Korea; 2 Department of Nanoscience & Nanotechnology, Kyungpook National University, Daegu, Republic of Korea; 3 Brain Science and Engineering Institute, Kyungpook National University, Daegu, Republic of Korea; 4 Department of Electrical and Electronic Engineering, Institute for IT Convergence, Hankyong National University, Anseong, Republic of Korea; University of California, Irvine, UNITED STATES

## Abstract

The Earth’s geomagnetic field (GMF) is known to influence magnetoreceptive creatures, from bacteria to mammals as a sensory cue or a physiological modulator, despite it is largely thought that humans cannot sense the GMF. Here, we show that humans sense the GMF to orient their direction toward food in a self-rotatory chair experiment. Starved men, but not women, significantly oriented toward the ambient/modulated magnetic north or east, directions which had been previously food-associated, without any other helpful cues, including sight and sound. The orientation was reproduced under blue light but was abolished under a blindfold or a longer wavelength light (> 500 nm), indicating that blue light is necessary for magnetic orientation. Importantly, inversion of the vertical component of the GMF resulted in orientation toward the magnetic south and blood glucose levels resulting from food appeared to act as a motivator for sensing a magnetic field direction. The results demonstrate that male humans sense GMF in a blue light-dependent manner and suggest that the geomagnetic orientations are mediated by an inclination compass.

## Introduction

Magnetoreceptive creatures—ranging from magnetotactic bacteria [1], to plants [2], to animals [3, 4] including certain birds, reptiles, insects, and mammals—are known to use the geomagnetic field (GMF) as a sensory cue for migration [3,4,5,6], short-distance movement [7–10], or as a physiological modulator [2], depending on the species. Evidence suggests that in magnetosensitive creatures, the GMF is sensed by either flavoprotein cryptochromes in the retina of the eyes or iron-containing biogenic magnetite in the head, which confers an inclination or polarity compass for navigation [3, 4]. Cryptochromes, in particular, need blue light (400–500 nm) when the GMF is sensed through a radical pair mechanism [11, 12], whereas light is not required for magnetite-mediated magnetoreception [3, 4]. For cryptochromes, light absorption by the prosthetic group, flavin adenine dinucleotide (FAD), triggers intramolecular electron transfer to form a spin-correlated radical pair whose spin-selective reactivity leads to magnetically sensitive reaction product yields [12]. In the process, a FAD-Trp radical pair is formed through photo-excitation of the FAD in its fully oxidized state, which has an absorption maximum of 450 nm and very weak absorption above 500 nm.

It is broadly accepted that humans are responsive to alternating magnetic fields, including low frequency pulsed magnetic fields used in medicine and clinical therapies [13, 14]. Such low frequency magnetic fields effectively promote wound healing, bone unification, pain reduction, blood circulation, and cancer treatment. In addition, a targeted pulse of transcranial magnetic stimulation facilitated working memory recall in humans [15]. By contrast, it is largely thought that the GMF, a static magnetic field, is not sensed by humans. A series of studies conducted by Baker [16,17,18], suggesting that human non-visual magnetic navigation by sensing the GMF through biogenic magnetite in the brain, attracted immense interest in human magnetic sense. However, the results have not been consistently replicated by others [19–21]. Nevertheless, some subsequent studies have also implied the existence of a magnetic sense in humans. Light sensitivity of the human visual system has been shown to be remarkably sensitive to the modulated direction of the GMF [22, 23]. Alternating magnetic fields significantly altered electroencephalogram signals in several brain regions, indicating the presence of magnetosensory evoked potentials [24, 25]. Moreover, human cryptochrome-rescued transgenic fruit flies showed light-dependent magnetoreceptive behavior, implying that the abundant cryptochromes in the retina of the human eye may elicit magnetoreception at a molecular level [26].

In ancestral human hunter-gatherer societies, the sustainability and prosperity of a community depended on successful residential movement in the direction of viable food sources [27]. Although primates receive sensory inputs such as sight, sound, and odor, from their surrounding sensory environments, to aid with successful residential movement, it is likely that they also developed an unrevealed spatial sense-memory system for long-range food search [28]. For optimal food foraging, primates, including humans, combine external common senses with food preferences determined by sight, taste, and smell of food, which is conformed and modulated unconsciously by integrative neural networks in the brain [29, 30]. As is well known, the brain almost exclusively uses glucose as its energy source and glucose-sensing neural circuits in the brain tightly control acute glucose homeostasis, feeding behavior, and energy expenditure [31].

## Results

### Human males can sense the geomagnetic field

**Fig 1:**
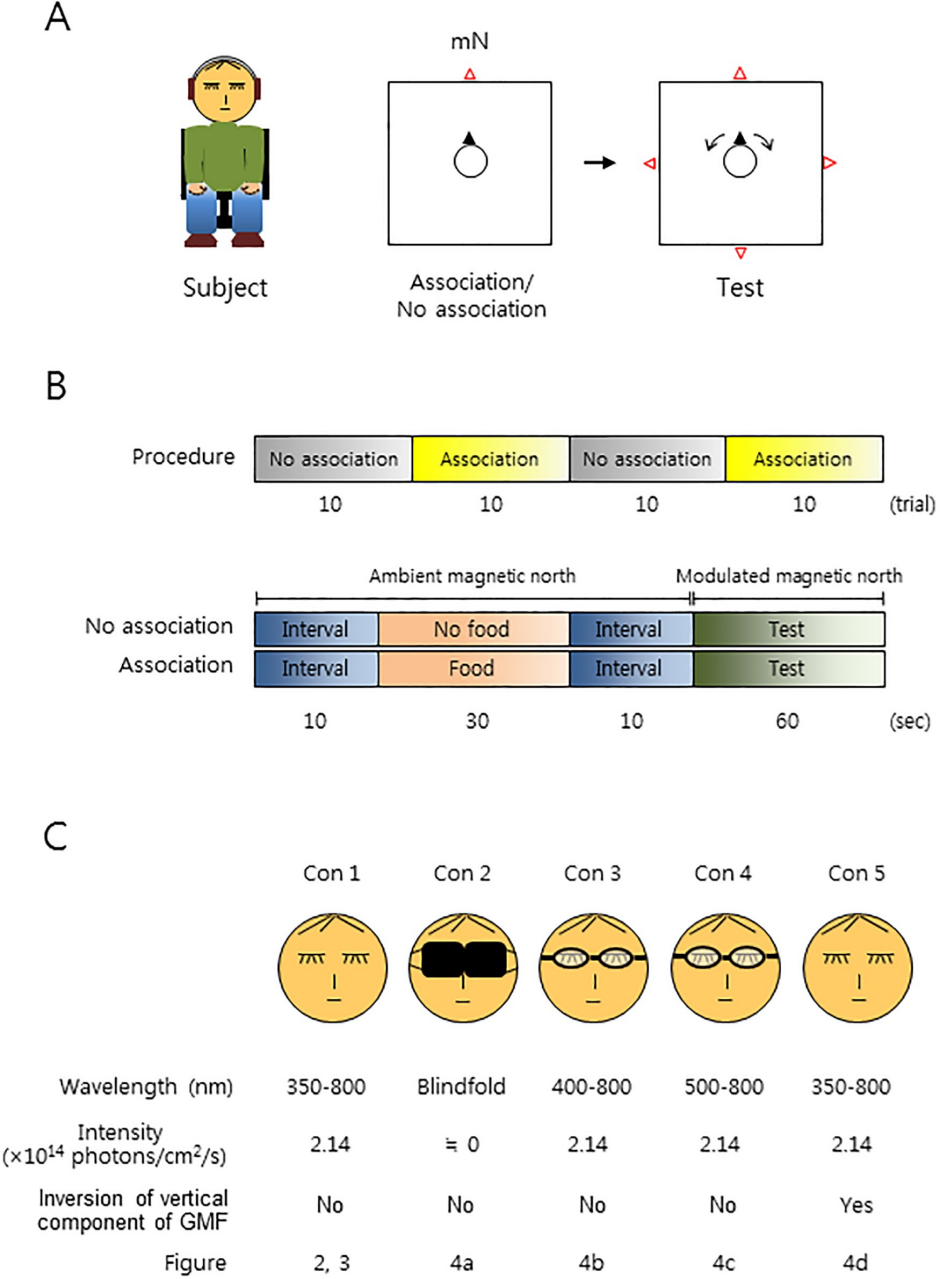
Schematic drawing of the geomagnetic food orientation assay. (**A**) A schematic drawing of a subject sitting on the rotatable chair and the direction of geomagnetic north during the food association/no association phase and the actual test. *Left*: Frontal view of the subject. Subjects had to wear earmuffs and close their eyes for the duration of the experiment. *Middle* and *right*: Top view of the subject facing toward the ambient geomagnetic north during the food association/no association phase, and four modulated geomagnetic norths, one of which was randomly provided to the subject during the test. Note that geomagnetic east instead of geomagnetic north was tested in a set of experiments in Fig 2. The subjects could choose to rotate clockwise or counterclockwise as indicated by the arrows during the test. mN, magnetic north; square, the vertical Helmholtz coils; circle, the subject; black closed triangle, the facing direction of the subject; red open triangle(s), the direction of ambient geomagnetic north (*middle*) or modulated geomagnetic north (*right*). (**B**) The procedure and timeline for the geomagnetic food orientation assay. Food was provided for ‘association’ trials only. (**C**) The light and magnetic field conditions for the experiments. The subjects had their eyes closed to avoid distraction and to prevent visual cues from aiding in the locating of the modulated magnetic north. Subjects were provided with either a full wavelength of light or light filtered by either a blindfold or filter glasses. Intensity, light intensity on the surface of the eyelids; Yes and No, inversion of the vertical component of the magnetic field was performed or not performed; Figure, the figure(s) in which the indicated experimental condition was provided.

**Fig 2:**
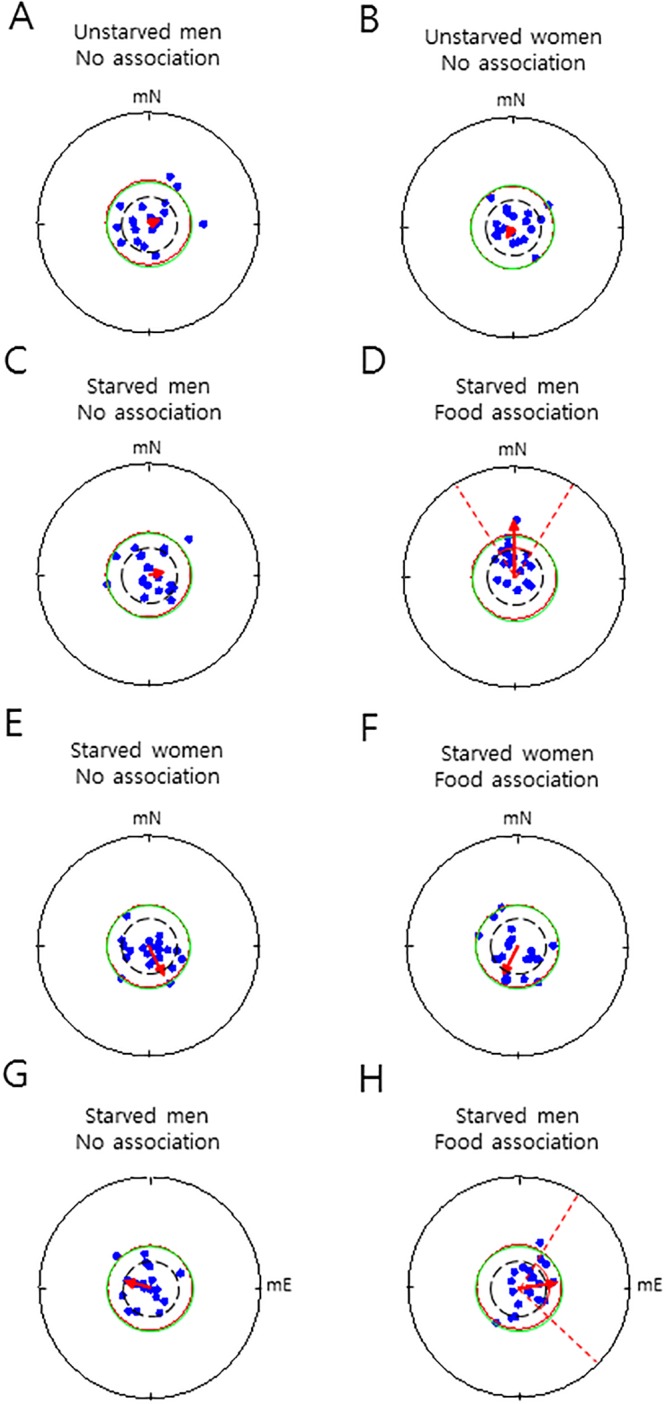
Geomagnetic field-sensitive food orientation in starved men. (**A**) and (**B**) The subjects had meals normally and were tested under the no association experimental condition. There was no notable magnetic north orientation in the unstarved men (A) and women (B). (A) *α* = 64.4°, *r* = 0.06, *P* = 0.43 (*V* test), *P* = 0.93 (Rayleigh test), *n* = 20. (B) *α* = 207.1°, *r* = 0.06, *P* = 0.64 (*V* test), *P* = 0.92 (Rayleigh test), *n* = 21. (**C**) and (**E**) No magnetic north orientation in the starved men (C) and women (E), when they were tested under the no association experimental condition. (C) *α* = 79.7°, *r* = 0.12, *P* = 0.45 (*V* test), *P* = 0.77 (Rayleigh test), *n* = 20. (E) *α* = 152.3°, *r* = 0.31, *P* = 0.96 (*V* test), *P* = 0.14 (Rayleigh test), *n* = 21. (**D**) and (**F**) A significant (D) and unremarkable (F) magnetic north orientation in the starved men and women, respectively, tested after the food association. (D) *α* = 350.0°, *r* = 0.51, *P* = 0.00043 (*V* test; 95% confidence interval, 326.9°–31.1°), *P* = 0.004 (Rayleigh test), *n* = 20. (F) *α* = 206.8°, *r* = 0.28, *P* = 0.96 (*V* test), *P* = 0.19 (Rayleigh test), *n* = 21. (**G**) and (**H**) An unremarkable (G) and a significant (H) magnetic east orientation in the starved men under the no association and the food association condition, respectively. (G) *α* = 285.8°, *r* = 0.24, *P* = 0.92 (*V* test), *P* = 0.33 (Rayleigh test), *n* = 20. (H) *α* = 83.2°, *r* = 0.34, *P* = 0.015 (*V* test; 95% confidence interval, 33.2°–133.2°), *P* = 0.09 (Rayleigh test), *n* = 20. Circular statistical analyses were performed using the *V* test and Rayleigh test for all experiments. Note that throughout the study, the *V* test was more appropriate to evaluate whether the subjects oriented toward the magnetic north (east) along which the food association/no association phase was conducted. In each circular diagram, each of the dots and arrow indicate the subject’s mean direction vector and group mean vector of the subjects, respectively. Throughout, the dashed circle, solid outer circle, and solid inner circle indicate the gradation of 0.25, the maximum value (i.e., 1.0) for the length of a subject’s mean vector, and the minimum length of the group mean vector needed for significance in the Rayleigh test (*P* = 0.05), respectively. mN, the modulated magnetic north; mE, the modulated magnetic east; *α*, group mean vector as clockwise degree; *r*, length of group mean vector. The dashed lines and the solid arc indicate the confidence interval and the minimum length of the group mean vector needed for significance in the *V* test (*P* = 0.05), respectively.

We speculated that it may be difficult to detect evidence of human magnetoreception, if it exists, under normal physiological states, as in prior studies. Adopting a modified chair experiment [24, 18], combined with a food-association paradigm and subject self-rotatory direction search, for the first time, we investigated magnetoreception in humans under starvation conditions. Subjects, who had been preconditioned to associate a direction with chocolate chips or not, depending on the experimental session, had to close their eyes, wear earmuffs, and were aligned by the experimenters facing toward the ambient magnetic north on a rotatable chair (Fig 1A and 1B). Then, in the absence of any other helpful cues, they were asked to sense and indicate the direction of the modulated magnetic north (see Materials and Methods). It is known that very low intensity light (approximately 3 × 10^−4^ lux = 1.28 × 10^8^ photons/cm^2^/s), which is comparable to the intensity of the light on moonless, starry nights, is sufficient for songbirds to use the GMF in nocturnal migration [32]. In humans, even a single photon of light can be detected by dark-adapted open eyes [33], while light transmission through the eyelids is 5.6% (red light) to 0.3% (blue light) [34]. Initially, unstarved subjects were tested without a food association (Fig 1C, *Con 1*), under full wavelength light from a light-emitting diode (Fig A in S1 Fig). Neither men nor women showed a notable orientation toward a direction (Fig 2A and 2B), suggesting that unstarved humans without a food association did not sense the magnetic north in this experimental condition. Next, starved subjects were examined, with or without a food association, under the same experimental conditions. To our surprise, only the food-associated men showed a significant orientation toward the modulated magnetic north, whereas women, regardless of the food association, did not show a significant orientation (Fig 2C–2F). A detailed analysis revealed that the food-associated men demonstrated significant orientation toward the ambient geomagnetic north and the three modulated magnetic norths (0°, 90°, 180°, and 270°) (Table 1). In a control experiment, the currents in the coils were made to flow in antiparallel directions so that the ambient magnetic north did not change during the test period, to which the subjects were blind (subjects were still asked to indicate the direction of the modulated magnetic north; see Materials and Methods). In this control experiment, the starved food-associated men did not orient toward the ambient, or *supposed*, magnetic north (Table 1). This result indicated that the orientation of the food-associated men (Fig 2D) was not determined by potential confounding factors, including heat or vibrations from the coils. In contrast to the starved food-associated men, unstarved food-associated men did not show a notable orientation (S2 Fig), indicating that fed humans may not sense the GMF. To confirm the potential magnetic sense in starved men, the subjects were asked to orient toward the modulated magnetic east instead of magnetic north in the same assay paradigm, where the food association was also towards the magnetic east. Consistently, only the starved food-associated men demonstrated a significant orientation toward the modulated magnetic east (Fig 2G and 2H). Thus, the results show that starved men, but not women, can sense the GMF and orient toward the magnetic direction with which food has been associated.

**Table 1:** Comparison of orientation in starved men between the normal and antiparallel current conditions.

Coil current	Modulated mN (°)	Parameters of circular statistics									
		*α*	*r*	*P* (*v*)	*P* (Rayleigh)	*n*	*α*	*r*	*P* (*v*)	*P* (Rayleigh)	*n*
		No-association					Food-association				
Normal	0	115	0.21	0.71	0.43	20	342	0.28	0.04 *	0.20	20
	90	179	0.14	0.82	0.67	20	9	0.30	0.03 *	0.17	20
	180	13	0.11	0.25	0.79	20	26	0.32	0.03 *	0.12	20
	270	63	0.27	0.22	0.23	20	359	0.29	0.03 *	0.19	20
Antiparallel	0 ^*a*^	168	0.31	0.97	0.15	20	164	0.29	0.96	0.20	20
	0 ^*b*^	82	0.22	0.42	0.40	20	325	0.12	0.26	0.74	20

^a^, ^b^ The degree for the modulated mN under the antiparallel current condition—the ambient GMF. Note that ^*a*^ and ^*b*^ represent virtually different mN to which direction vector for each trial was calculated relative.

* Statistically significant (*P* < 0.05)

In the normal condition, the test of a trial was performed under the ambient GMF (0°) or one of the three modulated magnetic norths (90°, 180°, and 270°). In the antiparallel current condition, the test was performed under the ambient GMF; however, subjects, who were blinded to the test condition, were still instructed to indicate the direction of the ambient/modulated magnetic north. The direction vector for each trial was calculated as a clockwise angle from the ambient magnetic north (normal-0° and ^*a*^), modulated magnetic north (normal-90°, -180°, -270°) or *supposedly modulated* magnetic north (^*b*^). *α*, group mean vector as a clockwise degree relative to the ambient/modulated magnetic north; *r*, length of the group mean vector; *P*(*v*), *P* value of the *v* test; *P*(Rayleigh), *P* value of the Rayleigh test; *n*, number of tested subjects

### Blue light and an inclination compass are crucial for human magnetoreception

**Fig 3:**
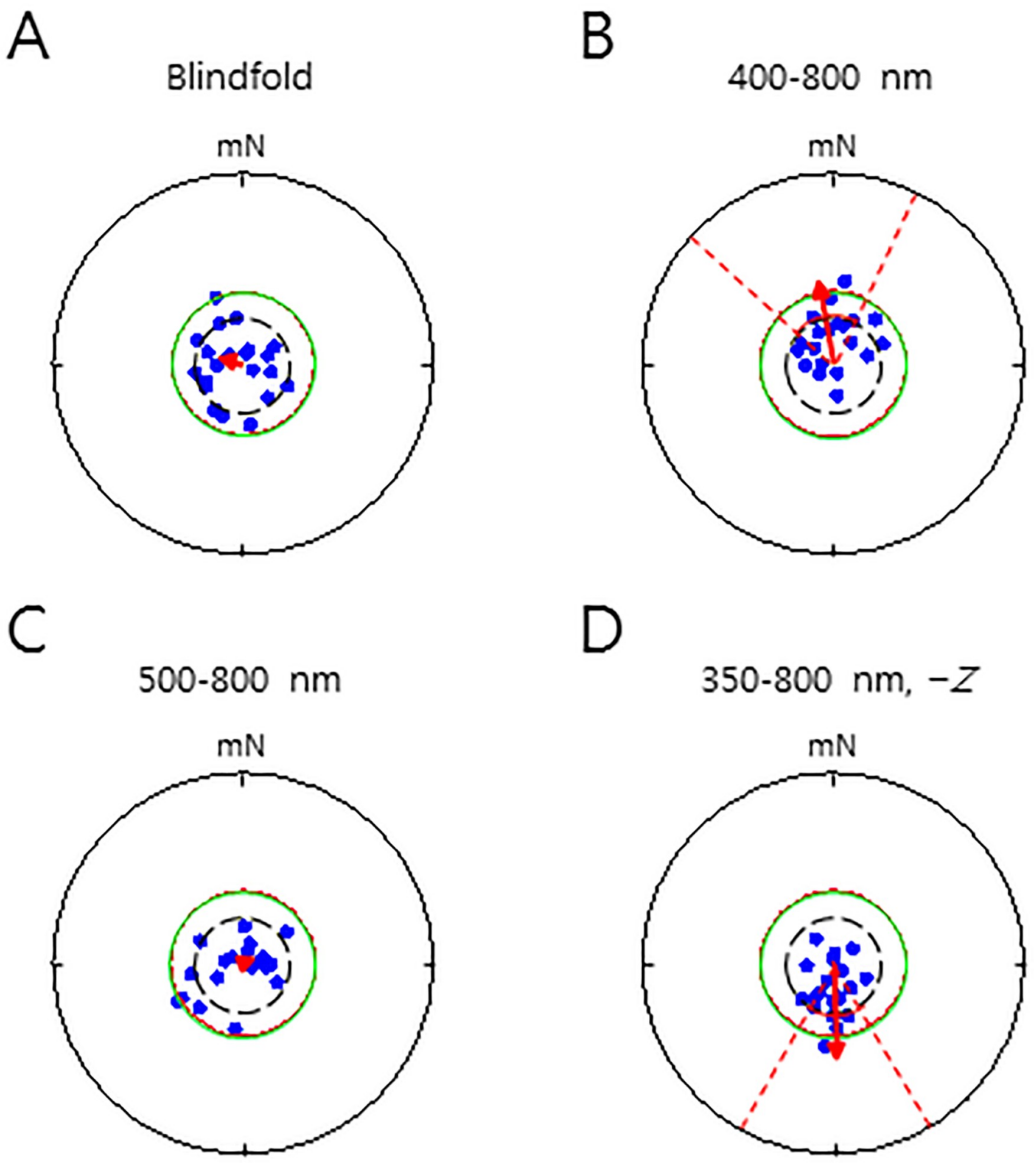
Reception of blue light by the eye is necessary for geomagnetic food orientation in starved men. The starved men were tested after the food association under different light conditions or a particular magnetic field condition. (**A**) and (**C**) An unremarkable magnetic north orientation under the blindfold (A) and the light (500–800 nm) (C). (A) *α* = 292.5°, *r* = 0.13, *P* = 0.52 (*V* test), *P* = 0.79 (Rayleigh test), *n* = 20. (C) *α* = 64.7°, *r* = 0.06, *P* = 0.44 (*V* test), *P* = 0.94 (Rayleigh test), *n* = 20. (**B**) A significant magnetic north orientation under the light 400–800 nm. *α* = 349.7°, *r* = 0.44, *P* = 0.003 (*V* test; 95% confidence interval, 311.4°–28.0°), *P* = 0.02 (Rayleigh test), *n* = 20. (**D**) A significant magnetic orientation toward magnetic south under inversion of the vertical component of the GMF (− *Z*) with the light (350–800 nm). *α* = 178.4°, *r* = 0.50, *P* = 0.00062 (*V* test; 95% confidence interval, 145.2°–211.6°), *P* = 0.006 (Rayleigh test), *n* = 20. The dashed lines and the solid arc indicate the confidence interval and the minimum length of the group mean vector needed for significance in the *V* test (*P* = 0.05), respectively. mN, the modulated magnetic north; *α*, group mean vector as clockwise degree; *r*, length of group mean vector.

The current results neither support nor negate cryptochrome or biogenic magnetite as a potential magnetoreceptor and inclination or polarity as a compass type, which underlie the mechanisms of magnetic orientation [3, 4]. Based on previous studies that suggest a relationship between light and magnetic sensitivity in humans [26,22,23], we examined the functional characteristics of the magnetic orientation in starved men. First, starved food-associated men wore a blindfold over their closed eyes, completely preventing light reception (Fig 1C, *Con 2*), and were asked to orient toward the modulated magnetic north. Surprisingly, blindfolding abolished the magnetic north orientation (Fig 3A), indicating that light penetration into the eyes was necessary for the GMF-sensitive orientation, and suggesting that a radical pair mechanism may underlie the magnetic orientation [11, 12]. In previous studies, blue light (400–500 nm) was necessary for the magnetic sense-related behaviors in a handful of light-dependent magnetoreceptive animals [7,35,36], and cryptochromes, potential magnetoreceptor proteins, were involved in magnetoreception as an active form in the retina [37]. To examine whether a particular wavelength of light was essential for the orientation in our study, the subjects were provided with filter glasses of either > 400 nm or > 500 nm (Figs B and C in S1 Fig) instead of the blindfold, in a subsequent set of experiments (Fig 1C, *Con 3* and *Con 4*). Orientation toward the modulated magnetic north was significant when light > 400 nm was provided, whereas light > 500 nm did not produce a notable orientation toward a direction (Fig 3B and 3C), showing that blue light was necessary for the geomagnetic orientation. In contrast, in control experiments, starved men who had not been associated with food did not show a clear orientation under the various light spectrum conditions (Figs A-C in S3 Fig), confirming that a food association was necessary for the starved men to demonstrate a magnetic orientation. These results show that blue light is necessary for the geomagnetic orientation. Given that inversion of the vertical component of the GMF can be used to indicate whether an inclination compass or a polarity compass is involved in magnetoreceptive orientation [4], we wanted to test this premise. Strikingly, inversion of the vertical component of the GMF under the full wavelength of light–the same light condition as in Fig 2D and 2H (Fig 1C, *Con 5*)–induced a significant orientation to the modulated magnetic *south* instead of the magnetic north, even though subjects were asked to indicate the direction of the magnetic north (Fig 3D). This result demonstrates that an inclination compass was used for magnetic orientation in men. Consistently, non-food-associated starved men did not show a noticeable orientation under the inversion condition (Fig D in S3 Fig). Thus, the results demonstrate that blue light and an inclination compass are crucial for magnetic orientation in starved men.

### Blood glucose activates sensing magnetic direction

**Fig 4:**
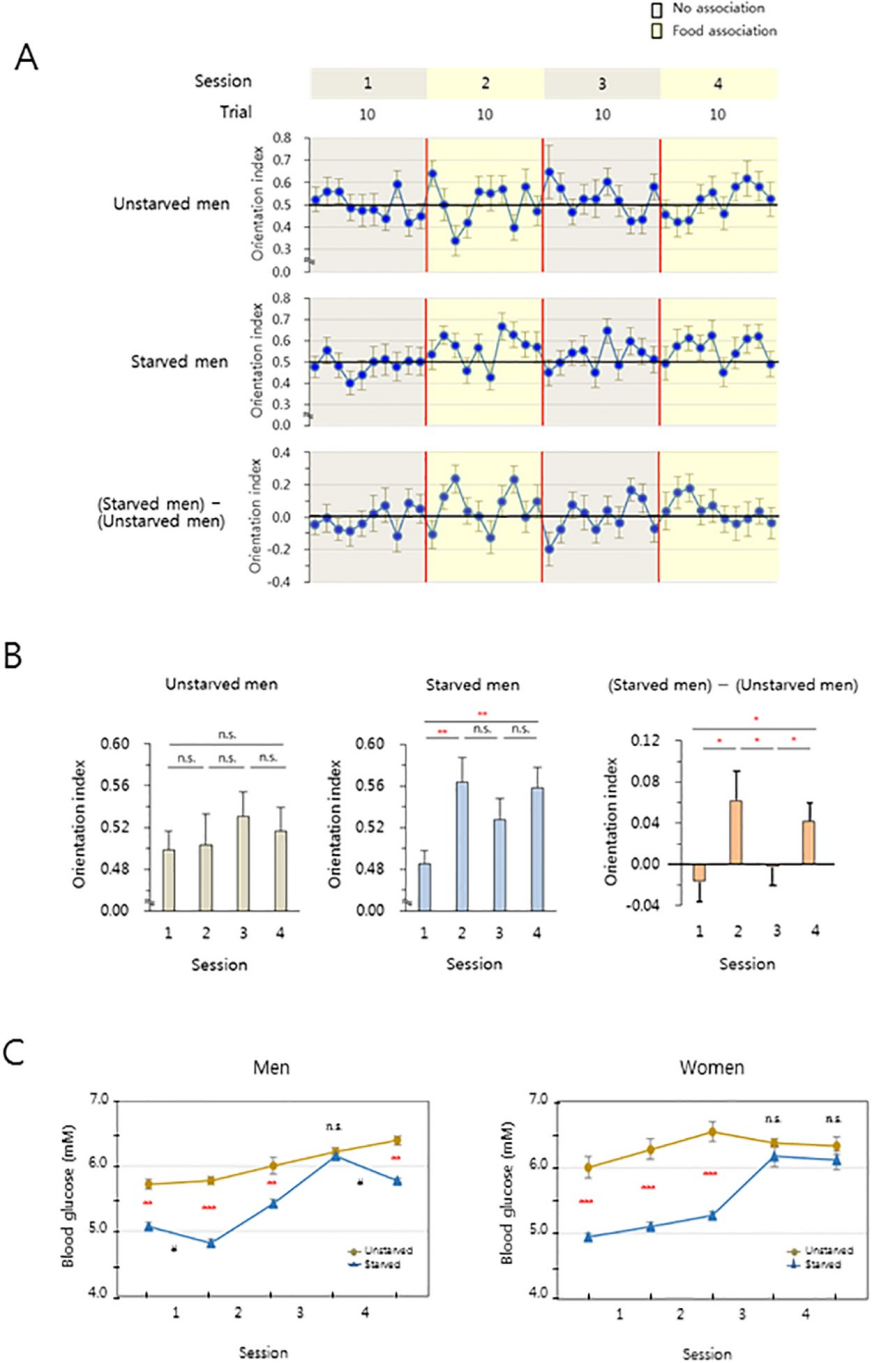
Glucose-motivated magnetic orientation in starved men. (**A**) Orientation profiles of unstarved men (*top*), starved men (*middle*), and (starved men–unstarved men) (*bottom*) from the tests in Fig 2A, 2C and 2D and Fig A in S2 Fig. The orientation index was calculated as [absolute value of (direction vector angle– 180°)] ∕ 180 and ranges 0 to 1. In each trial, the dot and error bar indicate the mean and standard error of the mean (SEM) of the 20 male subjects, respectively. (**B**) An analysis of the data in (A). (*left*, *center*) In each session, the values of mean and error bar denote the mean and SEM of dots in the corresponding session of a), respectively. (*right*) Mean and error bar of a session indicate the mean and SEM of the values calculated as [(orientation index value of a trial in the *center*)–(orientation index value of the same trial in the *left*)] in the session. Analysis of variance (ANOVA) test, n.s.: not significant. *, *P* < 0.05; **, *P* < 0.01. (**C**) Blood glucose levels during the magnetic north orientation assay in men (*left*) and women (*right*). In a separate set of experiments, blood glucose level at shortly before the first session and immediately after each session was determined in the same starved or unstarved men and women subjects. Student’s *t*-test, n.s.: not significant. #, *P* < 0.01; **, *P* < 0.0001; ***, *P* < 0.000001. Statistical values are presented as mean ± standard error of the mean (SEM).

Finally, we investigated the mechanism by which different magnetic orientations was manifested among starved men, unstarved men, and women irrespective of starvation. An analysis of raw data (Fig 2A, 2C and 2D, Fig A in S2 Fig) demonstrated that magnetic north orientation was remarkable only in the food association sessions in starved men (Fig 4A, *top*, *middle*), in consistent with the result (Fig 2D). The subtracted magnetic north orientation response was quick but not sustained in subsequent trials in both the food association sessions 2 and 4 (Fig 4A, *bottom*), suggesting that food was not a conditioning stimulus but rather a motivator for the orientation. This conclusion is supported by a previous study [38] that suggested almost constant GMF, which is ever present, as an unconditioned stimulus in the view of classical conditioning. In an additional analysis, comparison of the orientation index between the no association and food association sessions showed significant differences between adjacent sessions in starved men and the subtraction graph (Fig 4B, *center*, *right*). In particular, the significant difference between sessions 2 and 3 (Fig 4B, *right*), together with the immediate remarkable decrease in the orientation index of earlier trials in session 3 (Fig 4A, *bottom*), consistently indicated that the significant orientation in the food association sessions was a motivated, but not a conditioned, response. Based on previous studies [31] on neural circuits for control of glucose homeostasis, the chocolate chips’ nutrition facts of % Daily Value for men and women (sugars, 7.7% and 9.5%; total carbohydrates, 3.8% and 4.8%; respectively) (S1 Table) and the quick onset of food-motivated magnetic orientation (Fig 4A, *bottom*), we surmised that glucose from the food might be the motivator for the orientation. In a separate experiment to examine this hypothesis, subject’s blood glucose levels were measured shortly before the first session and immediately after each session. As expected, glucose level of subjects in the starved condition was slightly but significantly lower than that of subjects in the normally fed condition (*P* < 0.0001 or 0.000001) at most of the measurement time points in both men and women (Fig 4C). Interestingly, unlike in starved women (Fig 4C, *right*), a significant decrease in glucose level (*P* < 0.01) was observed in starved men during sessions 1 and 4 (marked as #) (Fig 4C, *left*), after and during which chocolate chips were provided, respectively. The results suggest that glucose may be key to the food-related motivation of starved men to orientate towards a certain magnetic direction during sessions 2 and 4.

## Discussion

Our findings show that, as a group, starved men can sense the GMF during food consumption and use it to identify the direction in which food can be obtained, even though significant individual orientation for food was not observed in most men. Importantly, unlike previous studies on human magnetoreception, magnetic field sensing and selective orientation through the subject’s voluntary rotational direction search and hand raising behaviors in the present study demonstrated a distinct and active magnetic responsiveness. As evidence for geomagnetic orientation in starved men, the antiparallel current condition that did not generate a *supposedly* modulated magnetic north, failed to produce a significant orientation in the subjects, unlike that seen under the normal condition (Table 1). It is conceivable that, if the subjects could detect the GMF direction, they should have oriented toward the ambient magnetic north in not only the normal condition but also the antiparallel condition. However, the result from the antiparallel condition suggests that the potential tendency of the subjects’ orientation toward the ambient magnetic north was not strong enough to override the influence of the pre-experiment instruction given to the subjects to indicate the direction of the modulated magnetic north. Particularly, the instruction may have led the subjects to expect a substantial extent of modulated magnetic north (~3/4 of total trials) besides the ambient GMF (~1/4 of total trials), thereby biasing them mostly away from alignment with the ambient magnetic north. In addition, the subjects showed a significant orientation toward all the modulated magnetic norths, including the ambient magnetic north (0°) under the normal condition (Table 1). In fact, the magnetic fields produced by the Helmholtz coil system in the present study were not entirely homogeneous and not the same as the GMF. Therefore, the results suggest that men could sense the ambient GMF north during the association period and used this information to identify the magnetic north direction of both the ambient GMF and the relatively inhomogeneous modulated magnetic fields generated by the Helmholtz coils during the test period.

The results show that blue light entering the eyes, the putative magnetoreception organ in humans, is essential for GMF-receptive food orientation. Given that birds demonstrated a magnetoresponsive orientation under light at wavelengths up to 571 nm [35], it is interesting that male subjects did not orient for wavelengths > 500 nm, warranting an investigation into the difference between birds and men. Moreover, there is increasing evidence that cryptochrome-based magnetoreception may occur even in the absence of continuous light [39]. In this regard, cryptochromes may serve as only one of the possible speculative magnetoreceptive molecules for the magnetic orientation. In addition, the underlying mechanism for magnetic responses remains to be established even though involvement of an inclination compass in magnetic orientation is generally consistent with what would be expected from the radical pair mechanism. Despite the possible cryptochrome-radical pair scenario, this scenario does not necessarily rule out the potential role of brain magnetite as either a combined contributor in the results or a surrogate for cryptochrome as a magnetoreceptor in unknown magnetic responses.

The male-specific magnetic orientation might have originated from prehistoric male ancestors who were dominantly responsible for gathering or hunting for food, and the varying level of individual orientation could be a diverging trait from the evolutionary process to date. Nevertheless, it cannot be ruled out that other geomagnetic responses may exist in women. Based on the tight correlation between the contrasting blood glucose level in men (a significant decrease in sessions 1 and 4) vs women (no notable decrease) and the significant difference in magnetic orientation, a cognitively demanding task [40], we can speculate a magnetosensitive sensory-motor process for the orientation despite absence of direct evidence for the link between food cue and the remarkable orientation in starved men. Similar to the increase in active cryptochromes in the retinal ganglion cells in migratory birds [37], men’s cryptochromes in the retinal ganglion cells [41] may be activated in function or for expression per se by the starvation and/or post-food glucose surge, which might promote magnetoreception rendering magnetic orientation for food. This scenario could be supported, at least in part, by a recent study in mice that demonstrated that fasting conditions confer sex-dependent differential cryptochrome expression in the liver, resulting in differing lifespans and aging between sexes [42]. However, whether retinal cryptochrome expression under the starvation condition is different between men and women has yet to be assessed. Directional information induced by the potential association between magnetic direction and increased blood glucose concentration or food taste, which might be formed during the association period [31], could be projected into head direction cells that discharged independent of the location and ongoing behavior of the subject [43], which may be critical for the subject’s search for food. A recent study showing that blind rats were able to find food in mazes using food-associated geomagnetic information from a head-mounted magnetic compass [44], possibly supports the notion. Future investigations using human eye cultures or eye organoids combined with spatio-temporal analysis of brain activity under modulated magnetic field may be helpful in obtaining insights on magnetoreception and the mechanistic process of magnetic orientation in humans.

The present study shows that blue-light dependent human magnetoreception occurs in the eyes in a manner that appears to involve the brain and glucose, whereas the magnetoreceptor and magnetoreception mechanism (radical pair or otherwise) remains to be established.

## Materials and methods

### Subjects

A total of 41 normal subjects with no physical disability or mental disorders, including claustrophobia, participated in the present study, similar to previous studies [22–25, 27]. Twenty men (19–33 years; mean age, 22 years and body mass index (BMI) 18–29 kg/m^2^; mean, 22 kg/m^2^) and 21 women (19–23 years; mean age, 20 years and BMI 18–29 kg/m^2^; mean, 21 kg/m^2^) completed the experiments, while 4 men and 3 women could not finish the experiments due to personal reasons. They were informed of the goals and general procedure of the study and were asked to indicate one of the four modulated magnetic north or east directions, depending on the experiment, as described in the geomagnetic orientation assay below. Before each experiment, the subjects were required to follow regulations for the study including short-term starvation (18–20 h; no food except pure water after dinner no later than 7 pm one day before the test day) according to a typical protocol [45, 46], no medicinal treatments, and normal sleep (for at least 6 h, between 10 pm the day before the test day to 8 am on the test day). Subjects who did not follow the regulations, according to assessment with a pre-experiment questionnaire, were not allowed to take the test. The study was approved by the Institutional Review Board of Kyungpook National University and all the procedures followed the regulations for human subject research.

### Modulation of GMF

In the testing room, the ambient GMF had a total intensity of 50 μT, inclination of 53°, and declination of −7° (Daegu city, Republic of Korea). To provide the subjects with different GMF-like magnetic fields, i.e., with the same total intensity and inclination, but different direction for magnetic north, a rectangular Helmholtz coil system used in our previous studies [10, 47] was double-wrapped and electrically-grounded with copper mesh shielding [48], and used to generate static magnetic fields as described [10, 47]. Briefly, the dimensions of the coils were 1,890 × 1,890 mm, 1,890 × 1,800 mm, and 1,980 × 1,980 mm, for the X, Y, and Z axes, respectively. The coil for the X-axis (north-south) was aligned with the true north, while the Y-coil (east-west axis) and Z-coil (vertical axis) were used to modulate the *Y* and *Z* components of the GMF, respectively. The subject was seated on a rotatable plastic chair without any metal component at the center of the coils and the subject’s head was positioned at the middle space of the vertical axis. To modulate the geomagnetic north, a pair of coils for each axis was supplied with DC from a power supply (E3631A; Agilent Technologies, USA). GMF was measured using a 3-axis magnetometer (MGM 3AXIS; ALPHALAB, USA) and the homogeneity of the GMF at the space for the subject’s head was measured as 95%. In a control experiment, the current flowed through each double-wound coil in antiparallel directions [48] to discern any artifacts such as heating or vibration from magnetic field effects. The testing room was shielded by a six-sided Faraday cage comprising 10-mm-thick aluminum plates, which was grounded during the entire experiment [49]. The electromagnetic noise was measured before the beginning and at the end of each experiment inside the coils, and the noise was attenuated by at least 200-fold from 500 Hz to a higher frequency; the magnetic component between 500 Hz and 1 kHz was measured with Fluxgate Mag670-R-100 (Bartington Instruments, UK) and the magnetic/electric components between 1 kHz and 1 MHz were measured using 3D NF Analyzer NFA 1000 (Gigahertz Solutions, Germany). The total intensities of alternating magnetic fields and electric fields, from 500 Hz up to 1 MHz, were no more than 15 nT and 60 μV/m, respectively. Between 1 and 100 MHz, the maximum intensities of magnetic/electric components were 0.005 nT (bandwidth of 10 kHz, Model 6511 for 1–5 MHz and HI-4433-HCH for 5–100 MHz; ETS-Lindgren, USA) and 0.001 nT (bandwidth of 10 kHz, EFS 9218; Schwarzbeck Mess-Elektronik, Germany) The 60 Hz power frequency magnetic field was no more than 4 nT (3D NF Analyzer NFA 1000; Gigahertz Solutions, Germany). All electronic devices were placed outside the Faraday cage during experiments. The temperature over the subjects was maintained as 25 ± 0.3°C (USB Data logger 98581; MIC Meter Industrial Co., Taiwan) by air circulation through the honeycomb on the ceiling of the Faraday cage.

### Geomagnetic orientation assay

All experiments were performed between 1 pm and 5 pm (local time, UTC + 09:00). Depending on the experiment, starved or unstarved subjects were tested individually. Prior to each experiment, the subjects were asked to remain with their heads facing the front without roll, yaw, or pitch, with eyes closed and their earmuffs on, and refrain from speaking during the experiment. In particular, they were asked to concentrate on sensing, if they could, the ambient geomagnetic north/east during the association period, and to use the sensed information for orientation toward one of modulated magnetic norths (i.e., 0°, 90°, 180°, or 270° clockwise relative to the ambient geomagnetic north)/east during the test period. Subjects were advised to avoid distracting thoughts and to instantly think “which direction is the modulated magnetic north/east?” whenever they were distracted during the test period. Seated on the rotatable chair, the subjects were stabilized for 3 min before the beginning of the first trial in the absence of any sensory cues, including visual, auditory, olfactory, and haptic cues. They were illuminated with a filtered/non-filtered diffused light-emitting diode light (350–800 nm), depending on the experiment, as indicated in Fig 1C. The light source was positioned directly above the subjects so that it provided no directional information. The blindfold and filter glasses were worn throughout an experiment, including the food association/no association and test period, when required. The blindfold was made of thick black fabric, which completely prevented light from entering the eyes. The home-made filter glasses used glass filters [10] (Tae Young Optics, Republic of Korea) and provided the eyes with particular wavelengths of light (400–800 nm, 500–800 nm; Spectrometer USB4000-UV-VIS, Ocean Optics, USA) (S1 Fig). Each experiment consisted of two repetitions of 10 sequential trials for ‘no association’ and ‘food association’ as depicted in Fig 1B, similar to previous studies that tested a reward-conditioned magnetic compass in pigeons [50] or chickens [51] under inhomogeneous and homogeneous magnetic field conditions, respectively. For the food association, a subject who faced toward the ambient geomagnetic north or east, depending on the experiment, was gently provided with a chocolate chip [52] (nutrient facts, S1 Table) on his/her right palm by an experimenter, and given 30 s to eat it. During no association trials, food was not provided during the association period. After a subsequent 10-s interval, the experimenter gently touched the subject’s right thenar area using a paper rod as the cue to start the test. A randomly selected modulated GMF, with one of the four magnetic north directions (i.e., 0°, 90°, 180°, or 270°), was provided 2 s before the cue for the test. For the 0°-modulated magnetic north (i.e., the ambient magnetic field), no current was conducted through the coils. Each of the modulated magnetic north directions was provided five times for no association sessions (20 total trials) and food association sessions (20 total trials) in the experiment. Subjects were informed of the direction before each experiment, including the antiparallel current condition experiment. Thus, the instruction led subjects to expect magnetic north changes during the test period even in the antiparallel current condition experiment, which was indistinguishable from the normal condition experiments for the subjects. With the touch cue, unlike a previous study [18], subjects were asked to freely rotate toward any direction (clockwise or anticlockwise) by themselves, which helped prevent potential dizziness from rotation, and then asked to try to sense the direction of the modulated magnetic north or east, depending on the experiment, during a 1 min period. When subjects determined the direction for the magnetic north or east, they stopped rotating to face toward the direction and lifted their right hand to indicate the direction to the experimenter. The direction was measured with the eyes of the experimenter who aligned the top of the subject’s head with a scale (10° intervals) on the walls of the Faraday cage. When the subjects did not indicate the direction within the 1 min period, the trial was not included in the data and was repeated (approximately 0.5% of trials). Before the subsequent trial, the subjects were gently rotated to face toward the ambient geomagnetic north or east, depending on the experiment, and then rested for 10 s. All experiments were performed in a double-blind fashion. The experimenter who conducted the orientation assay recognized whether a subject was blindfolded, wearing filter glasses, or was food-associated or not. However, the experimenter did not know the random magnetic field sequences that were controlled by the PC system. Another experimenter who analyzed the data did not know whether they were collected from men/women, starved/unstarved, or associated/not associated subjects. Thus, none of the experimenters were aware of all the subject information and data during the experiments and data analysis. In a separate experiment, subject’s blood glucose levels were determined by the experimenter who conducted the orientation assay, using a blood glucose meter (Accu-Chek Guide; Roche, Germany) [53], shortly before the first session and immediately after each session according to the manufacturer’s protocol. Each of the measurements took less than 1 min and data from the experiment were not included in any orientation assay results. All the subjects participated in all the experiments by interspersion with a random processing order of experiments, and experiments were scheduled with an interval of at least 3 days between experiments for each subject. After each experiment, the subjects were individually asked to answer a post-experiment questionnaire about whether they closed their eyes during the entire period of the experiment. In case a subject did not maintain closed eyes, the experiment was repeated (approximately 1% of experiments).

### Statistical analysis

To determine the significance of the subjects’ orientation behavior, circular statistics were performed using the *V* test and Rayleigh test [54] for each group orientation (Oriana 4; Kovach Computing Services, UK). The “mean direction vector” of each subject was determined by averaging the direction vectors of the 20 trials for association or no association. The “direction vector” for each trial was calculated as a clockwise angle for a subject’s facing direction from the modulated magnetic north during a test period in each trial. The angle of the mean direction vector for association or no association in each subject was used to calculate the group mean vector using the *r*-vector statistics of Oriana 4. The statistical parameters were as follows: *α*, group mean vector as clockwise degree; *r*, length of group mean vector; SEM, standard error of mean. In the *V* test, the mean vectors were flanked by their 95% confidence interval limits and statistical significance was set at *P* < 0.05. In the Fig 4, statistical analyses were performed by one-way analysis of variance (ANOVA) Tukey’s test or Student’s *t*-test using Origin software (San Clemente, CA, USA). Statistical values are presented as mean ± standard error of the mean (SEM). In all the analyses, *P* < 0.05 was regarded as significant.

## Supporting information

S1 Fig Spectra of the lights used in the experiments. (**A**)–(**C**) Spectrums of light-emitting diode lights that were filtered by nothing (A), filter glasses (> 400 nm) (B), and filter glasses (> 500 nm) (C). Dashed lines denote the cutoff points of the filters.
(PDF)

S2 Fig Absence of geomagnetic orientation in food-associated humans without starvation. (**A**) and (**B**) No significant geomagnetic orientation in the unstarved men (A) and women (B), tested after the food association. (A) *α* = 109.4°, *r* = 0.15, *P* = 0.62 (*V* test), *P* = 0.65 (Rayleigh test), *n* = 20. (B) *α* = 89.4°, *r* = 0.08, *P* = 0.50 (*V* test), *P* = 0.87 (Rayleigh test), *n* = 21. Circular statistical analyses were performed using the *V* test and Rayleigh test for all experiments. In each circular diagram, each of the dots and arrow indicate the subject’s mean direction vector and group mean vector of the subjects, respectively. The dashed circle, solid outer circle, and solid inner circle indicate the gradation of 0.25, the maximum value (i.e., 1.0) for the length of a subject’s mean vector, and the minimum length of the group mean vector needed for significance in the Rayleigh test (*P* = 0.05), respectively. mN, modulated magnetic north; *α*, group mean vector as clockwise degree; *r*, length of group mean vector.
(PDF)

S3 Fig Absence of geomagnetic orientation in starved men without food association. The starved men were tested after no association under different light conditions or a particular magnetic field. (**A**)–(**D**) An unremarkable magnetic north orientation under the blindfold (A), light (400–800 nm) (B), light (500–800 nm) (C), and the inversion of vertical component of the GMF (−*Z*) with the light (350–800 nm) (D). (A) *α* = 168.7°, *r* = 0.12, *P* = 0.77 (*V* test), *P* = 0.76 (Rayleigh test), *n* = 40. (B) *α* = 243.2°, *r* = 0.24, *P* = 0.75 (*V* test), *P* = 0.31 (Rayleigh test), *n* = 20. (C) *α* = 311.6°, *r* = 0.12, *P* = 0.31 (*V* test), *P* = 0.76 (Rayleigh test), *n* = 20. (D) *α* = 24.4°, *r* = 0.04, *P* = 0.59 (*V* test), *P* = 0.97 (Rayleigh test), *n* = 20. Circular statistical analyses were performed using the Rayleigh test and *V* test for all experiments. In each circular diagram, each of the dots and arrow indicate the subject’s mean direction vector and group mean vector of the subjects, respectively. The dashed circle, solid outer circle, and solid inner circle indicate the gradation of 0.25, the maximum value (i.e., 1.0) for the length of a subject’s mean vector, and the minimum length of the group mean vector needed for significance in the Rayleigh test (*P* = 0.05), respectively. mN, modulated magnetic north; *α*, group mean vector as clockwise degree; *r*, length of group mean vector.
(PDF)

S1 Table Nutrient facts for chocolate chips used in experiments. ^*a*^ Twenty chocolate chips were provided to a subject in an experiment. ^*b*^ The % Daily Value means how much a nutrient in a serving food contributes to a daily diet. 2,600 and 2,100 calories a day are used for general nutrition advice for South Korean men and women (19–29 years old), respectively [Ministry of Health & Welfare of Republic of Korea. Dietary reference intakes for Koreans 2015. The Korean Nutrition Society; 2016].
(PDF)
