# Correction: Blue light-dependent human magnetoreception in geomagnetic food orientation

**DOI:** 10.1371/journal.pone.0223635

**Published:** 2019-10-03

**Authors:** Kwon-Seok Chae, In-Taek Oh, Sang-Hyup Lee, Soo-Chan Kim

The caption for Table 1 incorrectly appears as the final paragraph of the “Human males can sense the geomagnetic field” subsection of the Results. Please see the complete, correct Table 1 caption here.

**Table 1:** Comparison of orientation in starved men between the normal and antiparallel current conditions. In the normal condition, the test of a trial was performed under the ambient GMF (0°) or one of the three modulated magnetic norths (90°, 180°, and 270°). In the antiparallel current condition, the test was performed under the ambient GMF; however, subjects, who were blinded to the test condition, were still instructed to indicate the direction of the ambient/modulated magnetic north. The direction vector for each trial was calculated as a clockwise angle from the ambient magnetic north (normal-0° and ^*a*^), modulated magnetic north (normal-90°, -180°, -270°) or *supposedly modulated* magnetic north (^*b*^). *α*, group mean vector as a clockwise degree relative to the ambient/modulated magnetic north; *r*, length of the group mean vector; *P*(*v*), *P* value of the *v* test; *P*(Rayleigh), *P* value of the Rayleigh test; *n*, number of tested subjects.

Coil current	Modulated mN (°)	Parameters of circular statistics									
		*α*	*r*	*P* (*v*)	*P* (Rayleigh)	*n*	*α*	*r*	*P* (*v*)	*P* (Rayleigh)	*n*
		No-association					Food-association				
Normal	0	115	0.21	0.71	0.43	20	342	0.28	0.04 ^*^	0.20	20
	90	179	0.14	0.82	0.67	20	9	0.30	0.03 ^*^	0.17	20
	180	13	0.11	0.25	0.79	20	26	0.32	0.03 ^*^	0.12	20
	270	63	0.27	0.22	0.23	20	359	0.29	0.03 ^*^	0.19	20
Antiparallel	0 ^*a*^	168	0.31	0.97	0.15	20	164	0.29	0.96	0.20	20
	0 ^*b*^	82	0.22	0.42	0.40	20	325	0.12	0.26	0.74	20

^*a*^, ^*b*^ The degree for the modulated mN under the antiparallel current condition—the ambient GMF. Note that ^*a*^ and ^*b*^ represent virtually different mN to which direction vector for each trial was calculated relative.

* Statistically significant (*P* < 0.05)
