# Supplementary figures and images for: Blue light-dependent human magnetoreception in geomagnetic food orientation
Source: PLoS One. 2019 Feb 14;14(2):e0211826. doi: 10.1371/journal.pone.0211826 (PMC6375564; doi:10.1371/journal.pone.0211826)

Figure S1. Spectra of the lights used in the experiments.

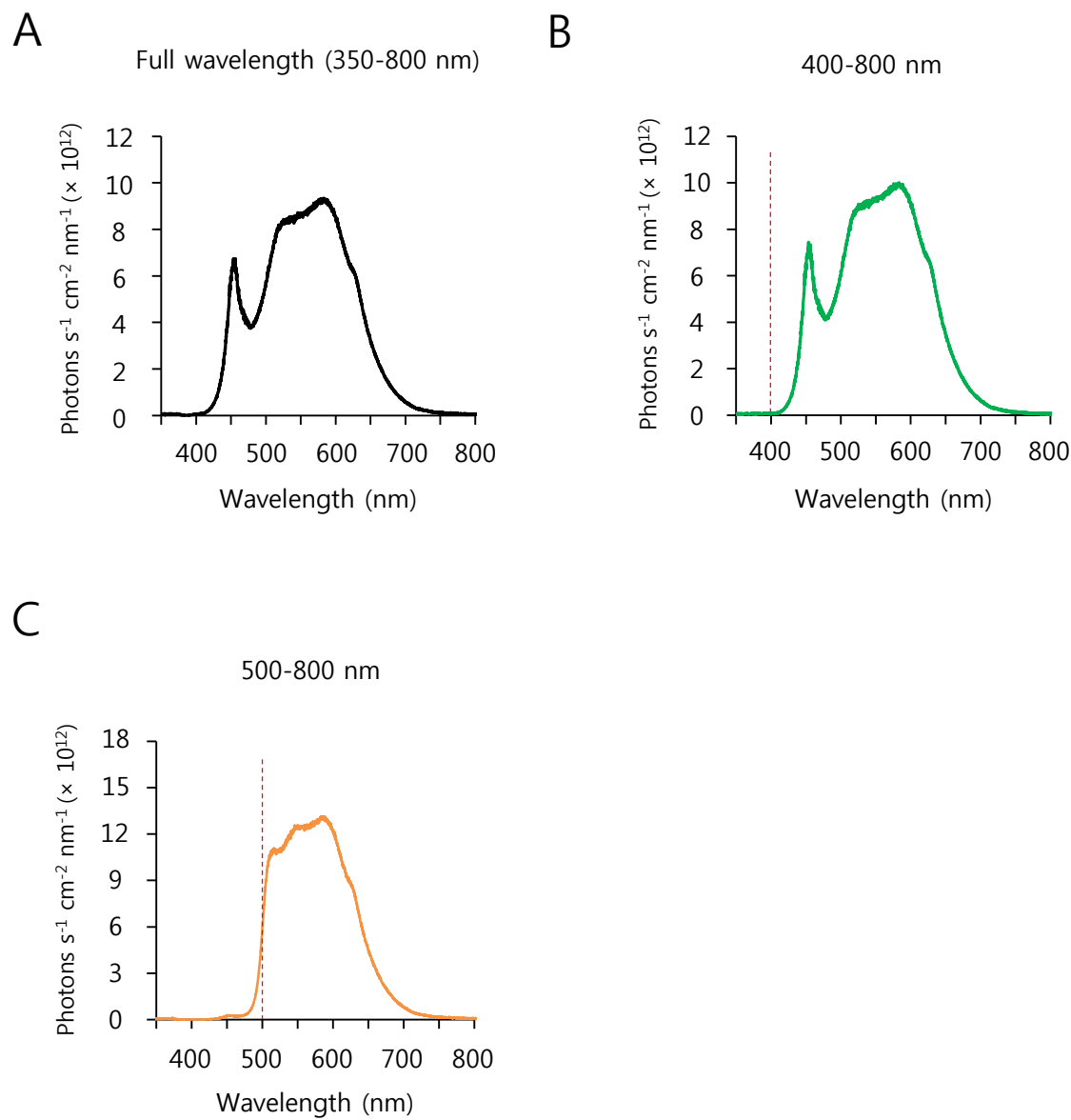

Supplement: S1 Fig — (A)–(C) Spectrums of light-emitting diode lights that were filtered by nothing (A), filter glasses (> 400 nm) (B), and filter glasses (> 500 nm) (C). Dashed lines denote the cutoff points of the filters. (PDF) [file pone.0211826.s001.pdf]

Figure S2. Absence of geomagnetic orientation in food-associated humans without starvation

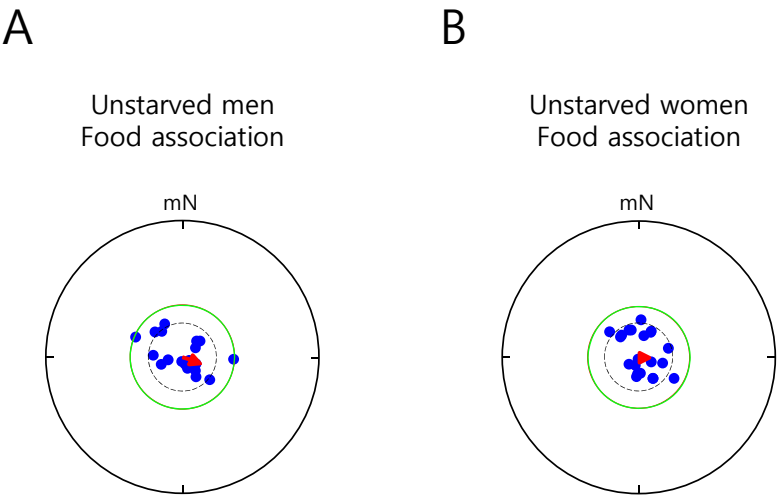

Supplement: S2 Fig — (A) and (B) No significant geomagnetic orientation in the unstarved men (A) and women (B), tested after the food association. (A) α = 109.4°, r = 0.15, P = 0.62 (V test), P = 0.65 (Rayleigh test), n = 20. (B) α = 89.4°, r = 0.08, P = 0.50 (V test), P = 0.87 (Rayleigh test), n = 21. Circular statistical analyses were performed using the V test and Rayleigh test for all experiments. In each circular diagram, each of the dots and arrow indicate the subject’s mean direction vector and group mean vector of the subjects, respectively. The dashed circle, solid outer circle, and solid inner circle indicate the gradation of 0.25, the maximum value (i.e., 1.0) for the length of a subject’s mean vector, and the minimum length of the group mean vector needed for significance in the Rayleigh test (P = 0.05), respectively. mN, modulated magnetic north; α, group mean vector as clockwise degree; r, length of group mean vector. (PDF) [file pone.0211826.s002.pdf]

Figure S3. Absence of geomagnetic food orientation in starved men without food association

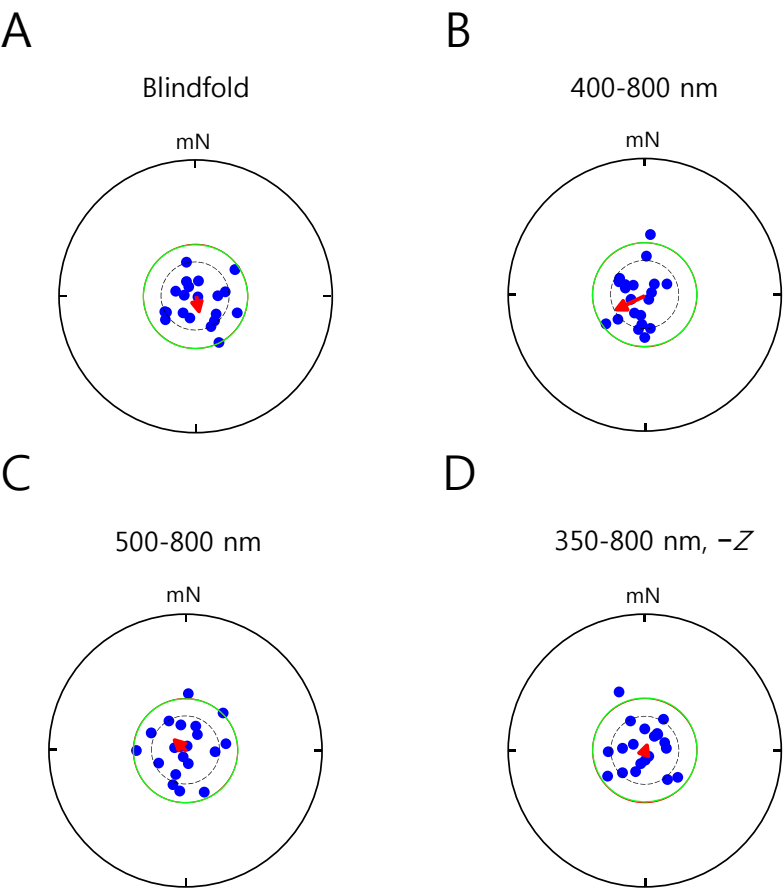

Supplement: S3 Fig — The starved men were tested after no association under different light conditions or a particular magnetic field. (A)–(D) An unremarkable magnetic north orientation under the blindfold (A), light (400–800 nm) (B), light (500–800 nm) (C), and the inversion of vertical component of the GMF (−Z) with the light (350–800 nm) (D). (A) α = 168.7°, r = 0.12, P = 0.77 (V test), P = 0.76 (Rayleigh test), n = 40. (B) α = 243.2°, r = 0.24, P = 0.75 (V test), P = 0.31 (Rayleigh test), n = 20. (C) α = 311.6°, r = 0.12, P = 0.31 (V test), P = 0.76 (Rayleigh test), n = 20. (D) α = 24.4°, r = 0.04, P = 0.59 (V test), P = 0.97 (Rayleigh test), n = 20. Circular statistical analyses were performed using the Rayleigh test and V test for all experiments. In each circular diagram, each of the dots and arrow indicate the subject’s mean direction vector and group mean vector of the subjects, respectively. The dashed circle, solid outer circle, and solid inner circle indicate the gradation of 0.25, the maximum value (i.e., 1.0) for the length of a subject’s mean vector, and the minimum length of the group mean vector needed for significance in the Rayleigh test (P = 0.05), respectively. mN, modulated magnetic north; α, group mean vector as clockwise degree; r, length of group mean vector. (PDF) [file pone.0211826.s003.pdf]
