# Supplementary material for: Blue light-dependent human magnetoreception in geomagnetic food orientation
Source: PLoS One. 2019 Feb 14;14(2):e0211826. doi: 10.1371/journal.pone.0211826 (PMC6375564; doi:10.1371/journal.pone.0211826)
Supplement: S1 Table — a Twenty chocolate chips were provided to a subject in an experiment. b The % Daily Value means how much a nutrient in a serving food contributes to a daily diet. 2,600 and 2,100 calories a day are used for general nutrition advice for South Korean men and women (19–29 years old), respectively [Ministry of Health & Welfare of Republic of Korea. Dietary reference intakes for Koreans 2015. The Korean Nutrition Society; 2016]. (PDF) [file pone.0211826.s004.pdf]

Table S1. Nutrient facts for chocolate chips used in experiments

**Serving size**	**20 chips[a] (27 g)**	**147 kcal**	
		% Daily Value[b]	
		Men	Women
**Total Fat**	7 g	10.0	12.4
Saturated Fat	5 g	23.8	29.5
Trans Fat	0.3 g		
**Cholesterol**	< 3 mg	0.5	0.7
**Total Carbohydrate**	17 g	3.8	4.8
Total Sugars	10 g	7.7	9.5
**Protein**	2 g	2.3	2.9
**Sodium**	30 mg	0.8	1.0
**Calcium**	37 mg	3.8	4.8
